# The association between occupational stress and depressive symptoms and the mediating role of psychological capital among Chinese university teachers: a cross-sectional study

**DOI:** 10.1186/s12888-014-0329-1

**Published:** 2014-11-30

**Authors:** Xue Shen, Yi-Long Yang, Yang Wang, Li Liu, Shu Wang, Lie Wang

**Affiliations:** Department of Social Medicine, School of Public Health, China Medical University, No.77 Puhe Road, Shenyang North New District, Shenyang, Liaoning China

**Keywords:** Depressive symptoms, Psychological capital, Occupational stress, University teachers

## Abstract

**Background:**

Depression is a major public health problem that affects both individuals and society. Previous studies report that university teachers are particularly susceptible to high levels of occupational stress and depressive symptoms. The aims of this study were to explore the association between occupational stress and depressive symptoms in a group of university teachers, and assess the mediating role of psychological capital between these variables.

**Methods:**

A cross-sectional study was performed between November 2013 and January 2014. Teachers from six universities were randomly sampled in Shenyang. The Center for Epidemiologic Studies Depression Scale, effort-reward imbalance scale, and psychological capital questionnaire (PCQ-24), as well as questions about demographic and working factors, were administered in questionnaires distributed to 1,500 university teachers. Completed questionnaires were received from 1,210 participants. Hierarchical linear regression analysis was used to examine the mediating role of psychological capital.

**Results:**

In the present study, 58.9% (95% CI (Confidence Intervals): 56.1% to 61.7%) of university teachers had a CES-D score equal to or above the cut-off of 16. Both effort–reward ratio (ERR) and scores of over-commitment were positively associated with depressive symptoms, whereas psychological capital was negatively associated with depressive symptoms among university teachers. Psychological capital partially mediated the relationship between occupational stress and depressive symptoms.

**Conclusions:**

Among Chinese university teachers, occupational stress may be a risk factor for depressive symptoms, whereas psychological capital might be protective against depressive symptoms. Our results suggest that college administrators could support the development of psychological capital in their staff to alleviate depressive symptoms.

## Background

Depression is a disease that can affect anyone, and is defined as feelings of guilt, worthlessness, helplessness and hopelessness. Its symptoms include loss of appetite, depressed mood, or disturbed sleep [1]. According to the World Health Organization (WHO), depression will likely be the second leading cause of disability worldwide by 2020 [2]. Depression affects a relatively large proportion of individuals. In Korea, the prevalence of depressive symptoms is reported as 26.1% for men and 28.7% for women [3]. Similarly, a study in China reported that nearly one third of the general population suffered from depressive symptoms [4]. Depression rates may differ amongst different professions, with some occupations having increased risk. In America, 12.2% of Iowa farmers reported high level of depressive symptoms [5]. Identifying at risk occupational groups is important because previous studies indicate that depression can increase the risk of obesity, suicidal ideation, burnout, and job turnover [6-8]. It may also negatively affect job satisfaction, quality of life, and well-being [9,10].

In China, rapid development of higher education and expansion of enrollment in universities has meant that the competition between colleges and universities is becoming increasingly intense. Teachers are put under pressure with substantial teaching responsibilities and scientific research work. This pressure may contribute to mental health problems. Recently, exploring factors associated with depression has become an expanding area of research. Studies suggest that workload and an adverse psychological environment at work significantly predict depression among teachers [7,11,12]. Moreover, studies in various populations suggest that social anxiety, work-related stress, and stressful life events may contribute to depression [13-15].

Occupational stress has therefore come to be considered by many as a risk factor for depressive symptoms [16-18].

Occupational stress can be assessed using the effort-reward imbalance model (ERI), which focuses on reciprocity of extrinsic effort, intrinsic effort, and reward [19]. Kinman and colleagues reported that high effort and low rewards within the ERI model significantly predicted negative outcomes, including psychological distress, physical symptoms, and low job satisfaction among UK university employees [20]. Teaching may be a profession that is particularly associated with high stress [8,21], as it requires speedy decision-making, and balancing the demands of and relationships with superiors, colleagues, and students. Moreover, there are additional pressures that include promotion, performance appraisal, and redundancy. This is reflected in a study of 17 Australian universities, where researchers found that 43% of academic staff and 37% of non-academic staff experienced occupational stress [22].

Teachers in every culture consider the individual differences of their students, teaching each according to their ability to learn. Thus, teachers are tasked with the important duty of aiding in the healthy development of students, as well as being required to provide quality intellectual education. However, teachers in China have additional pressure from the cultural adherence to Confucian principles. There are extremely strong social expectations that every teacher follow the philosophy of Confucius.

Some scholars have attempted to identify positive resources for combating occupational stress, such as perceived organizational support and psychological capital (PsyCap) [23,24]. PsyCap is proposed as a measurable high-order construct, comprising four state-like psychological resources: self-efficacy, hope, optimism, and resilience [25]. According to Luthans and colleagues, PsyCap goes beyond human capital, social capital, and financial capital, and can be measured, developed, and leveraged with various desirable results [26]. Specifically, when employees have high PsyCap, they are equipped with extra resources to handle their work tasks, expect good things to happen, quickly “bounce back” after setbacks, and are more hopeful about negative situations. Positive PsyCap, as an emerging core construct, has attracted the attention of many researchers in various fields, including private businesses (relating to their employees), medicine (relating to both staff and patients), and education (relating to students) [10,16,25,27]. Previous research has shown that PsyCap is positively associated with job performance, job satisfaction, and well-being [25,28], but negatively associated with depression, burnout, and staff turnover [16,24,29]. According to Luthans and colleagues, PsyCap plays a mediating role in the relationship between a supportive organizational climate and staff performance [30]. Lin and colleagues reported that PsyCap was a mediator between perceived organizational support and job burnout among employees of international tourist hotels in China [31]. Furthermore, Cheung and colleagues have suggested that PsyCap is a moderator between emotional labor (the requirement that employees display appropriate emotions to clients and others) and job satisfaction [32]. Therefore, previous research suggests that PsyCap may be particularly significant in the development of depression in occupational settings. Hence, we herein examine the potential mediating effects of PysCap on the posited association between occupational stress and depressive symptoms among university teachers.

The literature discussed above suggests that occupational stress and PsyCap are associated with depressive symptoms. However, there is a lack of understanding of potential variables that may reduce the impact of occupational stress on depressive symptoms. The findings of Liu and colleagues suggest that PsyCap plays a mediating role in the relationship between occupational stress and depressive symptoms among Chinese female physicians. Therefore, we hypothesize that PsyCap might have the mediating effect on the relationship between occupational stress and depressive symptoms among Chinese university teachers. This has not previously been investigated.

The main objectives of our study are to a) assess the prevalence of depressive symptoms among Chinese university teachers; b) explore associations between occupational stress, PsyCap, and depressive symptoms; and c) examine the mediating role of PsyCap in the relationship between occupational stress and depressive symptoms.

## Methods

### Research design and sample

A cross-sectional survey of health status among university teachers was conducted from November 2013 to January 2014 in Shenyang city, the capital of Liaoning province and the center of higher education in Northeast China. Universities in Liaoning province are mainly located in Shenyang city, with the total number of full-time teachers reaching 24,888 (according to Shenyang Statistical Information Net, 2013) [33]. Six universities (two comprehensive universities and four specialized universities focusing on medicine and architecture) were randomly selected and 25% of full-time teachers were randomly sampled from each selected university. Self-administered questionnaires were distributed to 1,500 teachers after obtaining written informed consent, and answers were received anonymously. A total of 1,210 effective responses were obtained (effective response rate: 80.7%). This study was approved by the Committee on Human Experimentation of China Medical University, and study procedures were in accordance with ethical standards.

### Demographic and working characteristics

Demographic characteristics collected included gender, age, marital status, education, and professional position. Marital status was categorized as single/widowed/divorced and married/cohabiting. Education was categorized as bachelor/master/doctor. Professional position was classified into assistant/lecturer/associate professor/professor. Participants were categorized as having chronic disease if they responded ‘yes’ to ever receiving a diagnosis of any listed disease (e.g., hypertension, diabetes, gout, cardiovascular disease, chronic gastritis, back pain, and arthritis).

### Measurement of depressive symptoms

Depression was measured by the Chinese version of the Center for Epidemiologic Studies Depression Scale (CES-D) [1]. This scale consists of 20 items related to characteristic symptoms and behaviors of depression, with each item rated from 0 to 3. The CES-D has been widely used in Chinese populations with good reliability and validity [4,34]. The total score ranges from 0 to 60, and CES-D ≥ 16 indicates that respondents may be more likely to be depressed [35]. In the present study, Cronbach’s alpha was 0.891. Additionally, confirmatory factor analysis (CFA) on a single factor model with twenty scale items was conducted to support validity of the depression questionnaire among university teachers (χ^2^/df = 3.925, CFI = 0.959, GFI = 0.951, TLI = 0.950 and RMSEA = 0.049).

### Measurement of occupational stress

We used the effort-reward imbalance (ERI) model to assess occupational stress experienced by teachers. The Chinese version of the ERI questionnaire, developed by Professors Li and Yang, is considered a reliable and valid instrument for measuring psychosocial stress at work [36]. The 23-item ERI questionnaire comprises three subscales: effort (6 items), reward (11 items) and over-commitment (6 items). Responses to “effort” and “reward” items were scored on a five-point scale, while responses to “over-commitment ” items were scored from 1 to 4. Effort–reward ratio (ERR) was calculated using a predefined algorithm that quantifies the degree of mismatch between high cost and low gain, with a correction factor of 0.5454 [37]. An ERR ratio greater than 1.0 indicates that the individual’s reward was not matched by the effort he/she made. An ERR ratio of less than 1.0 indicates the opposite. In this study, Cronbach’s alpha coefficients for effort, reward, and over-commitment subscales were 0.870, 0.898, and 0.725, respectively.

### Measurement of psychological capital (PsyCap)

PsyCap was measured with the Chinese version of the 24-item Psychological Capital Questionnaire (PCQ) [25]. The PCQ consisted of four dimensions: self-efficacy, hope, resilience, and optimism. Each of the four dimensions comprised six items, rated from 1 (strongly disagree) to 6 (strongly agree). Higher values indicated higher levels of experienced PsyCap. The PCQ has been widely used, and demonstrates adequate reliability and validity in multiple samples [25,38].

In the present study, a confirmatory factor analysis (CFA) was performed to check for the fit of the scales with the data. We began by fitting this model with six items for each facet (i.e., self-efficacy, hope, resilience, and optimism) and then fit each of the four dimensions to the higher-order PsyCap. The second-order factor model showed a good fit with the data (χ^2^/df = 3.671, GFI = 0.944, CFI = 0.971, TLI = 0.964, and RMSEA = 0.047). For the total scale, the Cronbach’s alpha coefficient was 0.949. Cronbach’s alpha coefficients of self-efficacy, hope, resilience, and optimism were 0.905, 0.904, 0.818 and 0.745, respectively.

### Statistical analysis

Differences in continuous variables were examined using the Student’s t-test and one-way ANOVA, and the significance level was set at p < 0.05 (two-tailed). For the prevalence of depressive symptoms, participants scoring 16 or higher were defined as depressed. Pearson’s correlation analysis was used to assess correlations between study variables. Hierarchical linear regression analysis was used to explore the effects of groups of independent variables on depressive symptoms, and the fit of the model was assessed with R^2^.

We tested the mediating role of PsyCap according to the theory of Baron and Kenny [39]. Accordingly, the following conditions should be satisfied: (1) the independent variable (ERI) is significantly associated with the dependent variable (depressive symptoms); (2) the independent variable is significantly associated with the mediator (PsyCap); (3) the mediator is significantly associated with the dependent variable after controlling for the effect of the independent variable. If the effect of the independent variable on the dependent variable shrinks upon the addition of the mediator to the model it is a partial mediator. If the independent variable does not affect the dependent variable when the mediator is added to the model then full mediation is established.

All study variables were centralized before being entered into the hierarchical linear regression model for the purpose of avoiding multicollinearity [40]. In Block 1, the control variables were added, including gender, age, marital status, and education. In Block 2, the independent variable (ERI) was added. In Block 3, the mediator (PsyCap) was added. The Sobel test was performed to calculate the significance of the mediating effect. All analyses were conducted using SPSS 17.0 for Windows. Statistical significance was defined as p < 0.05 (two-tailed).

## Results

### Participant characteristics

Table 1 shows the basic characteristics of the participants and the prevalence of depressive symptoms within the demographic categories. Respondents included 513 men (42.4%) and 697 women (57.6%), whose ages ranged from 24 to 62 years (mean 39.15 years, SD = 8.02). The prevalence of depressive symptoms was 58.9% (95% CI: 56.1% to 61.7%). Occupational stress (ERR > 1) was observed in 22.3% of university teachers. Teachers aged between 31 and 40 years old had significantly higher levels of depressive symptoms than other age groups (p < 0.05). Those with chronic disease had higher CES-D scores than those without (p < 0.05). Professors had lower CES-D scores than all others (p < 0.05). Differences in CES-D scores for gender, marital status, and education categories were not statistically significant (p > 0.05).

**Table 1:** **Demographic characteristics and differences in depressive symptoms**

**Variables**	**N**	**%**	**CES-D (Mean ± SD)**
Gender			
Males	513	42.4	18.63 ± 9.78
Females	697	57.6	18.37 ± 9.11
Age			
≤30	174	14.4	16.37 ± 8.60
31-40	565	46.7	19.48 ± 8.95**
41-50	367	30.3	18.12 ± 10.14
>50	104	8.6	17.84 ± 9.70
Marital status			
Single/widow/separated	186	15.4	17.72 ± 8.26
Married/cohabitation	1024	84.6	18.62 ± 9.59
Education			
Bachelor	168	13.9	19.47 ± 10.64
Master	557	46.0	18.77 ± 8.95
Doctor	485	40.1	17.80 ± 9.42
Professional position			
Assistant	105	8.7	17.82 ± 9.23
Lecturer	493	40.7	19.11 ± 8.97
Associate professor	445	36.8	19.19 ± 9.84**
Professor	167	13.8	15.17 ± 8.86
Chronic disease			
No	739	61.1	16.97 ± 8.74
Yes	471	38.9	20.85 ± 9.90**

CES-D: the Center for Epidemiologic Studies Depression Scale.

**p < 0.01 (two-tailed).

### Correlations between study variables

The means, standard deviations, and Pearson correlation analyses of continuous variables (age, CES-D, ERR, over-commitment, and PsyCap) are presented in Table 2. Results revealed that all variables were significantly correlated with each other except age (which only correlated with PsyCap), and these correlations were in the expected directions. Specifically, ERR and over-commitment were positively correlated with depressive symptoms, and negatively correlated with PsyCap. PsyCap was negatively correlated with depressive symptoms.

**Table 2:** **Means**, **standard deviations** (**SD**), **and correlations between continuous variables**

**Variables**	**Mean**	**SD**	**1**	**2**	**3**	**4**	**5**
1. Age	39.15	8.02	1				
2. CES-D	18.48	9.4	−0.009	1			
3. ERR	−0.18	0.25	0.041	0.463^**^	1		
4. Over-commitment	14.42	3.71	0.011	0.382^**^	0.654^**^	1	
5. PsyCap	4.26	0.78	0.077^**^	−0.461^**^	−0.442^**^	−0.326^**^	1

CES-D: the Center for Epidemiologic Studies Depression Scale; ERR: effort–reward ratio.

PsyCap: psychological capital.

**p < 0.01 (two-tailed).

### The mediating role of PsyCap in the association between occupational stress and depressive symptoms

Results of the hierarchical linear regression analysis to explore the mediating role of PsyCap are provided in Table 3. After adjustment for gender, age, marital status, and educational level, the independent variables ERR and over-commitment were added in block 2, before PsyCap was entered into block 3 of the model. The results revealed that both ERR and over-commitment were positively associated with depressive symptoms, explaining 22.6% of the variance in the data. However, PsyCap was negatively associated with depressive symptoms. Standardized regression coefficients (β) reduced with the inclusion of PsyCap, accounting for an additional 7.6% of the variance (p < 0.05). Therefore, PsyCap played a partial mediating role in the association of depressive symptoms with ERR (from β = 0.372 to β = 0.242; Sobel test, z = 9.73, p < 0.001) and over-commitment (from β = 0.148 to β = 0.135; Sobel test, z = 9.24, p < 0.001).

**Table 3:** **Results of the hierarchical linear regression analysis**

**Variables**	**Depressive symptoms**		
	**Block 1 (β)**	**Block 2 (β)**	**Block 3 (β)**
Gender	−0.025	−0.045	−0.054^*^
Age	−0.021	−0.026	0.001
Marital status	0.048	−0.02	−0.036
Dummy_e1	0.054	0.04	0.001
Dummy_e2	0.066^*^	0.072^**^	0.031
ERR		0.372^**^	0.242^**^
Over-commitment		0.148^**^	0.135^**^
PsyCap			−0.314^**^
F	1.578	52.088^**^	67.085^**^
Adjusted R^2^	0.002	0.228	0.304
△R^2^	0.007	0.226^**^	0.076^**^

Dummy_e1: Master vs. Doctor; Dummy_e2: Bachelor vs. Doctor; ERR: effort/reward ratio; PsyCap: psychological capital.

*p < 0.05, **p < 0.01 (two-tailed).

R^2^ (R-squared, the coefficient of determination): percentage of the sample response variation in dependent variable that is explained by the independent variable.

## Discussion

The present study explored the associations between occupational stress, PsyCap, and depressive symptoms, and further examined the mediating role of PsyCap. A large sample of teachers from comprehensive and specialized universities was studied, with a high response rate of 80.6%. The ratio of male to female participants was 1:1.36, which was similar to the value (1:1.3 to 1:1.4) obtained in other studies of university teachers in China [21,41,42]. Thus, our study population seems to be representative and supports generalizations made from the study.

We found a total prevalence of depressive symptoms of 58.9%, which is much higher than that reported in Korean employees (26.1% for men and 28.7% for women) [3], French nurse managers (33%) [43], and a Chinese general population sample (33.3%) [4]. However, it is similar to that of Chinese male correctional officers (61.4%) [44], and lower than that of Chinese doctors (65.3%) [45]. Therefore, our results support the assertion that Chinese university teachers might suffer from high rates of depressive symptoms. It is possible that the transformation of the education system and the strict management system of Chinese universities places an immense pressure on university staff in China, which could result in depressive symptoms [9,46]. Further cultural pressures, such as traditional Chinese thought (Confucianism), may instill particularly high expectations on teachers from parents and society. They are crucially responsible to impart both moral and intellectual knowledge, and are required to continuously absorb new knowledge to keep up with the high pace of modern technological information. Some teachers might become burned out, unable to cope with so much pressure, and so fall into a state of depression. Our results suggest that there is a great need for college administrators to develop and implement effective measures to alleviate teachers’ depressive symptoms and improve their mental health.

Additionally, our study found that both ERR and over-commitment were positively correlated with depressive symptoms, which is consistent with previous findings [10,47]. Occupational stress has been identified as a risk factor for depressive symptoms as it may influence mental health, quality of life, and job satisfaction [9,11,48]. In the present study, 22.3% of university teachers were categorized as experiencing occupational stress (ERR score >1), which is lower than reported for UK university employees (49%) [49] and teachers in New Zealand (26%) [50]. Various reasons for the experience of occupational stress have been suggested. Gillespie and colleagues reported that notable sources of stress for university staff included lack of recognition and reward [46]. Abouserie stated that university academic staff members were required to fulfill many competing responsibilities, such as teaching, researching, attending seminars, seeking funding, and writing papers, and 14.7% of the sample studies fell into the serious stress categories [51]. For university teachers, the level of occupational stress may affect their work efficiency and interpersonal relationships. It may be easy for them to develop a low mood, which to some extent develops into depressive symptoms. In addition, our results revealed that occupational stress was negatively correlated with PsyCap, which motivated us to explore the indirect effect of occupational stress in predicting depressive symptoms. Together, our results encourage further research and the development of preventative techniques to alleviate occupational stress and depressive symptoms among university teachers.

PsyCap is an important concept of organizational behavior, and is considered as a positive resource to combat destructive emotions, stress, burnout, and work–family conflict [25,52,53]. Consistent with this, in the present study PsyCap was found to be negatively associated with depressive symptoms among Chinese university teachers. Teachers with higher levels of PsyCap might have more confidence and exert greater effort in the pursuit of success (self-efficacy), preserve the will to accomplish a teaching task or goal (hope), bounce back from adversity or failure with positive psychological capacity (resilience), and have positive expectations and attributes regarding outcomes (optimism) [25]. Our results further support the suggestion that PsyCap might be a positive psychological resource for teachers to relieve depressive symptoms.

Besides providing additional support for previous findings, the major contribution of our findings is to highlight that occupational stress might affect risk for depressive symptoms in university teachers via a mediating mechanism of PsyCap. In other words, teachers who faced more occupational stress would be more likely to experience lower levels of PsyCap, which in turn would lead to higher levels of depressive symptoms. Empirical investigation shows that PsyCap can be developed using web-based training interventions [54,55]. Importantly, there is a great need for college administrators to support the development of PsyCap, to reduce occupational stress and improve quality of life among university teachers.

Our findings have theoretical and practical implications for human resources management and performance management. In theory, the present study revealed that PsyCap might be a positive resource for combating occupational stress and depressive symptoms. PsyCap is a core construct that organizations can invest in and develop in their workforce to achieve veritable, sustained growth and performance [30]. Practically, our results should highlight to college administrators the high prevalence of depression in university teachers. There is an urgent need for them to ensure a balance between effort and reward, establish fair performance appraisal systems, and improve social support systems and work environments. Furthermore, our study provides a new perspective for researchers to integrate the positive psychological resources of self-efficacy, hope, optimism, and resilience to relieve depressive symptoms. To improve self-efficacy, college administrators should provide more opportunities for teachers to develop their professional skills and career interests, and construct fair systems of rewards and punishment to boost the confidence of teachers. To enhance hope amongst teachers, college administrators should help staff formulate reasonable development goals, dividing large goals into manageable units and encouraging them to consider multiple pathways to accomplish each subgoal. For instance, small-group discussion may be an effective method of problem solving [56]. In this way, teachers’ hope can be increased, which in turn might enhance the level of self-efficacy. To improve optimism, supervisors should motivate employees to attribute positive events to personal, permanent, and pervasive causes, interpret negative events in terms of external, temporary, and situation-specific factors, and to seek available opportunities [57]. When employees are confident that they can identify and plan to overcome obstacles their expectations of achieving goals is increased. As for resilience, positive emotions have been empirically shown to enhance resilience in the face of negative events. College administrators should help teachers make plans to solve problems and overcome obstacles with available resources [55,58]. Furthermore, the psychological capital intervention (PCI) training model has been developed and used for online participants in developing PsyCap [54,59].

Several limitations of the present study must be taken into consideration when interpreting the results. First, this study mainly relied on self-report of participants, which risks response bias from negative affect or social desirability. Second, the list of potential diseases was rather limited. We enumerated several kinds of common chronic diseases, but some diseases such as addictions were not assessed. Third, generalizations from our data should be made with caution since the sample comprised only a small proportion of all teachers in China, and so may not be representative of all Chinese teachers or of teachers in other countries. Fourth, we only explored associations between occupational stress, PsyCap, and depressive symptoms, and examined differences in basic demographic variables. Other factors associated with depressive symptoms should be investigated in further research. Finally, as this study was cross-sectional, we are unable to draw conclusions about cause and effect. Future studies could incorporate a longitudinal design to increase the power of the findings. Indeed, we must acknowledge that reverse causation may explain the results with depressive symptoms determining levels of PsyCap and ERI (depressive symptoms–PsyCap–ERI). This model deserves further research attention.

## Conclusions

In our sample of Chinese university teachers, there was a high prevalence of depressive symptoms (score of 16 or above on the CES-D). Both extrinsic stress (ERR) and intrinsic stress (over-commitment) were positively associated with depressive symptoms, whereas PsyCap was negatively associated with depressive symptoms. PsyCap partially mediated the effect of occupational stress on depressive symptoms. Since PsyCap can be measured and developed, investing interventions to enhance PsyCap may provide new approaches to improve mental health and work efficiency amongst teachers.

## Abbreviations

CES-D: The center for epidemiologic studies depression scale
PsyCap: Psychological capital
ERI: Effort-reward imbalance
ERR: Effort–reward ratio
CI: Confidence interval
